# Isthmic tubal ectopic pregnancy from a partial molar pregnancy: A case report and literature review

**DOI:** 10.1016/j.crwh.2025.e00736

**Published:** 2025-07-22

**Authors:** Richard Q. Vuong, Molly Hurd, Zeynep Tek, Nicole Brzozowski

**Affiliations:** a Undergraduate Medical Education, Robert Larner, MD, College of Medicine at the University of Vermont, Burlington, VT 05405, USA; b Department of Obstetrics and Gynecology, Danbury Hospital, Danbury, CT 06810, USA

**Keywords:** Tubal ectopic pregnancy, Molar pregnancy, Gestational trophoblastic disease, p57, MRI

## Abstract

While tubal ectopic pregnancies and molar pregnancies are relatively common, tubal molar pregnancies are exceptionally rare, with an estimated incidence of 1.5 per 1,000,000 pregnancies. Molar pregnancies are at risk for developing malignant gestational trophoblastic neoplasia (15–20 % for complete hydatidiform moles and 0.5–6 % for partial hydatidiform moles) and warrant robust diagnostic workup to determine appropriate management. Presented here is a case of an isthmic tubal ectopic pregnancy secondary to a partial hydatidiform mole; additionally, a literature review through PubMed discusses all tubal molar pregnancies reported within a 5-year period.

A 41-year-old woman (gravida 2, para 0, aborta 1) at 6 weeks and 5 days of gestation presented to the emergency department from the outpatient clinic because prenatal ultrasound did not show an intrauterine pregnancy and her a β-hCG level was 17,913 mIU/mL. Due to concern for ectopic pregnancy, she underwent diagnostic laparoscopy, which revealed an unruptured right isthmic tubal ectopic gestation. The specimen was removed and sent for histological evaluation, which confirmed a partial hydatidiform mole that stained positive for p57; the finding was supported by molecular studies. The patient was discharged the same day and follow-up β-hCG levels were monitored weekly until undetectable. The literature review found that 13 tubal molar pregnancies had been reported between 2019 and 2024.

Histopathologic examination of gestational trophoblastic disease should be supported by ancillary studies such as immunohistochemical, flow cytometry, and molecular analyses to ensure accurate diagnosis. Magnetic resonance imaging could offer a way to preoperatively diagnose molar ectopic pregnancy in select hemodynamically stable patients.

## Highlights

• Ectopic pregnancies secondary to hydatidiform molar gestations are overdiagnosed.
• Immunohistochemistry should support the diagnosis of molar ectopic pregnancy.
• Magnetic resonance imaging may provide a preoperative diagnosis of molar ectopic pregnancy.
• Thirteen tubal molar pregnancies were reported in the literature from September 2019 to March 2024.
• Monitoring of β-hCG levels and contraception counseling are critical after molar pregnancy.

## 1 Introduction

Hydatidiform moles (HM), also called molar pregnancies, are a category of gestational trophoblastic diseases (GTD) and subclassified as a complete mole or a partial mole. Complete hydatidiform moles (CHM) result from fertilization of an ovum lacking maternal chromosomes, most commonly by a single sperm, leading to a karyotype of 46 XX [1]. Partial hydatidiform moles (PHM) occur when a haploid ovum is fertilized by two sperm or a diploid sperm, resulting in a triploid genome: 69 XXX, 69 XYY, or 69 XYY [2]. HM typically develops within the uterine cavity, but, on rare occasions, can occur ectopically (i.e. outside the uterine cavity). In the UK, the incidence of true ectopic molar pregnancy is 1.5 per 1,000,000 births [1]. Differentiating between CHM and PHM is imperative, as the risk for malignant gestational trophoblastic neoplasia (GTN) is much higher for CHM than PHM. To highlight the importance of robust diagnostic criteria, and discuss potential pre-operative diagnostic imaging modalities, this report describes a case of ectopic partial molar pregnancy and reviews the literature on tubal molar pregnancies within the PubMed database within a five-year period.

## 2 Case Presentation

A 41-year-old woman (G2, P0010) at 6 weeks and 5 days of gestation, based on last menstrual period, was referred from the maternal-fetal medicine clinic to the emergency department when a dating ultrasound failed to identify an intrauterine pregnancy and laboratory tests showed a β-hCG level of 17,913 mIU/mL. Transvaginal ultrasound visualized multiple, subserosal fibroids of the posterior (9.2 × 6.14 × 7.62 cm) and anterior (3.91 × 3 × 4.66 cm) wall, causing uterine cavity distortion, and a normal, small amount of echolucent fluid in the posterior cul-de-sac. The patient denied pelvic pain, abdominal pain, and vaginal bleeding. She was on the last day of a seven-day course of nitrofurantoin 100 mg for *Enterococcus faecalis* urinary tract infection.

Physical exam revealed a soft, nontender uterus with fibroids at approximately 14–15 weeks of gestation with no vaginal bleeding. Vital signs were stable, and laboratory results showed normal complete blood count and electrolyte panel. A repeat β-hCG test remained elevated, at 16,491 mIU/mL. Rh immunoglobulin was not administered as the patient was Rh-positive. Due to concern for ectopic pregnancy, the team proceeded with diagnostic laparoscopy.

The procedure was uncomplicated with an estimated blood loss of 5 mL. Upon manipulation of the fibroid uterus, a 2 × 3 cm unruptured isthmic tubal ectopic pregnancy was identified in the right fallopian tube. Bilateral ovaries and the left fallopian tube appeared normal. A right salpingectomy was performed in the standard fashion using the Maryland LigaSure to clamp, cauterize, and cut the mesosalpinx in serial segments to excise the ectopic pregnancy. The specimen was sent for histopathological analysis. The patient was discharged home the same day.

**Fig. 1:**
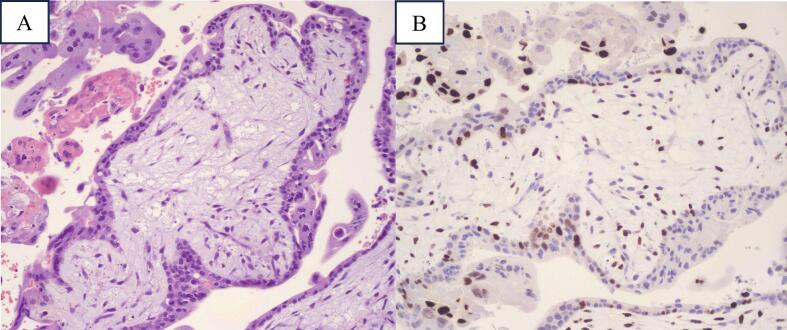
Histological findings of the case. A. Hematoxylin & eosin staining showing hydropic chorionic villi associated with trophoblastic proliferation. B. P57 staining showing positive signaling throughout the specimen, consistent with partial hydatidiform mole.

Histopathological examination revealed hydropic chorionic villi with associated trophoblastic proliferation (Fig. 1A) and positive p57 staining (Fig. 1B), consistent with partial hydatidiform mole. Complete hydatidiform moles are characterized by absence of p57 staining, while partial hydatidiform moles and non-molar pregnancies express p57 [3,4].

At a follow-up visit at the out-patient OBGYN clinic, the patient reported minimal vaginal bleeding and well-controlled post-operative pain with ibuprofen 600 mg every 6–8 h. β-hCG tests were performed weekly until β-hCG reached <5 mIU/mL, then obtained again in one month, when the level below the detectable threshold. The patient was counseled on contraception, avoiding pregnancy until β-hCG testing remained negative for one month, and that it was safe to attempt future pregnancy as desired after appropriate follow-up.

## 3 Literature Review

Using the PubMed database, articles related to the topic published between September 2019 and March 2024 were identified using the keywords “Molar ectopic pregnancy” and “molar tubal pregnancy”. Articles that discussed single case studies of tubal molar pregnancies, confirmed by histology, were included. Of the 54 articles identified, 13 met the inclusion criteria. One article was excluded because the patient was post-menopause; one article was excluded because it was not in the English language; one article was excluded because it was a meta-analysis; one article was excluded because it was a retrospective review; four articles were excluded because the location of the ectopic molar pregnancy was not tubal; and the remaining articles were excluded because they were not inherent to the search.

Thirteen cases were reported in the literature from 2019 to 2024 on tubal molar pregnancies (Table 1) [3,[5], [6], [7], [8], [9], [10], [11], [12], [13], [14], [15], [16]]. The mean age of diagnosis of tubal molar pregnancy was 30 years, with the predominant presenting symptoms being abdominal pain (8/13 cases, 61 %) and vaginal discharge, bleeding, or spotting (8/13 cases, 61 %) and less commonly amenorrhea (2/13 cases, 15 %), nausea and vomiting (3/13 cases, 23 %), and pelvic pain (2/13 cases, 15 %). On ultrasound examination, a left adnexal mass was present in 23 % (3/13) of patients, a right adnexal mass in 61 % (8/13), a thickened endometrium in 15 % (2/13), and hemoperitoneum in 8 % (1/13). Surgical intervention occurred in all cases, and when sent for histological evaluation, 46 % (6/13) were identified as CHM, 31 % (5/13) as PHM and 23 % (3/13) as other GTD. The predominant choice for follow-up was serial blood tests, namely β-hCG monitoring, though frequency varied. Of the five cases that reported the frequency of β-hCG follow-up, four reported weekly checks, and one reported every two weeks. Of the thirteen cases, four (28 %) reported GTD that needed follow-up with methotrexate treatment.

**Table 1:** Review of all tubal molar ectopic pregnancies from 1 September 2019 to 1 March 2024.

Author, Year	Study Design	Clinical Aspects	Ultrasound Findings	Treatment	Histological Examination	Follow-Up
Adhikari et al., 2023 [6]	Case Report	30-year-old **β-hCG**: 67,565 mIU/mL **Presenting Symptom:** lower abdominal pain, vaginal spotting, amenorrhea. **PMH**: G2P2, cesarean section	Empty uterus, left adnexal mass	Left partial salpingectomy	Partial hydatidiform mole	Weekly β-hCG for 6 months post surgery
Athanasiou et al., 2022 [3]	Case Report	50-year-old **β-hCG**: 83,346 **Presenting Symptom**: abdominal pain PMH: G2P1, Crohn's disease	left adnexal mass, 67 × 25 mm Hemoperitoneum	Exploratory laparoscopy and left salpingectomy	Complete Hydatidiform mole	Weekly serum β-hCG monitoring
Ayyash et al., 2022 [7]	Case Report	22-year-old **β-hCG**: 23,833 mIU/mL **Presenting Symptom**: abdominal pain, nausea and vomiting, vaginal bleeding **PMH**: G2P1001, History of and positive for *Trichomonas vaginalis, Chlamydia trachomatis, and Neisseria gonorrhoeae*	Complex fluid in the right adnexa measuring 1.16 × 1.20 × 1.1 cm with circumferential color Doppler flow.	Laparoscopic right salpingectomy	Partial hydatidiform mole	Lost to follow up 2 months after procedure Repeat β-hCG was <10 mIU/mL
D'Asta et al., 2022 [8]	Case Report & Literature Review (2010−2020)	27-year-old **β-hCG**: 590 mUI/mL **Presenting Symptom**: Right pelvic pain, vaginal bleeding **PMH**: G2P0, left tubal ectopic pregnancy treated with left salpingectomy	Unilocular cyst, 15 mm in size adjacent to the right ovary. Anechoic fluid and peripheral vascular ring. Endopelvic free fluid, 26 mm in size.	Admitted, daily monitoring & serial BHCG measurements. BHG continued to rise, right laparoscopic salpingectomy.	Incomplete invasive vesicular mole with extrauterine implants	Serial monitoring of β-hCG level; β-hCG = 0 one month after surgery Continued β-hCG monitoring every 2 weeks until 3 consecutive months' negative levels Whole body CT scan
Dollinger et al., 2021 [9]	Case Report	40-year-old **β-hCG**: 68 K IU/L **Presenting Symptom**: Abdominal pain **PMH**: G6P6	Hemoperitoneum	Right salpingectomy for ruptured ectopic pregnancy	Complete hydatidiform mole	Referred to gynecologic oncology, Diagnosed with stage 2 GTN. Received 11 cycles of MTX before reaching disease remission.
Figueiredo et al., 2022 [10]	Case Report	20-year-old **β-hCG**: 49.728 IU/L **Presenting Symptom**: nausea, vomiting and vaginal discharge **PMH**: G1P0, miscarriage 2 months prior	Irregular, poorly defined endometrium, thickness of 34 mm uterine cavity filled by blood clots Determined to be interstitial ectopic molar pregnancy	Left cornual resection with ipsilateral salpingectomy	Complete Hydatidiform mole Low-risk GTN	Weekly β-hCG monitoring, β-hCG =0 at one month post surgery CT scan of chest, abdomen and pelvis-discovered metastasis Methotrexate therapy
Hasan et al., 2021 [11]	Case Report	35-year-old **β-hCG**: 30,000 units/mL **Presenting Symptom**: lower abdominal pain, amenorrhea **PMH**: G3P2a	4.5 cm × 3 cm swelling adjacent to the right ovary with a gestational sac containing a viable gestational sac with active heartbeats and body motions	Right salpingectomy	Partial Hydatidiform mole	Serial β-hCG monitoring (frequency not reported)
Hosseini et al., 2023 [12]	Case Report	29-year-old **β-hCG:** 23,400 pg/mL **Presenting Symptom:** None **PMH**: G0P0, IVF treatment, endometriosis, primary infertility, laparoscopy of cystadenoma	TVUS: endometrial thickness 7 mm, 27 × 21 mm hyper- hetero- echoic lesion with peripheral vascularization	3 × 4 cm mass adherent to the right ovary and fallopian tube excised	Partial hydatidiform mole	Weekly β-hCG monitoring until 3 consecutive tests came back negative
Najib et al., 2023 [13]	Case Report	34-year-old **β-hCG**: 14,000 IU/L **Presenting Symptom**: vaginal bleeding, right lower abdominal pain. **PMH**: G4P3	7-week gestational sac in the right adnexa and moderate free fluid with an internal echo in the pelvic cavity; however, no fetal pole was observed	Right salpingectomy	Tubal choriocarcinoma Stage 2 GTN	Six sessions of chemotherapy β-hCG level monitoring (frequency not reported)
Shen et al., 2023 [14]	Case Report	22-year-old **β-hCG**: 13,996 IU/L **Presenting symptom:** Abdominal pain **PMH**: G2P1	Heterogenous mass, right adnexa, 22 × 21 mm, contained a 14 × 9 × 11 mm gestational sac and 6 mm embryo with active heartbeats. No intrauterine gestational sac	Right salpingectomy	Complete hydatidiform mole	Serial β-hCG monitoring (frequency not reported) Follow up ultrasound
Swamy et al., 2023 [15]	Case Report	24-year-old **β-hCG**: 150,045 ng/mL **Presenting Symptom:** hyperemesis and pelvic pain, vaginal spotting **PMH**: G2P2	Left adnexal heterogeneous hyperechoic mass *MRI also performed	Left salpingectomy	Complete hydatidiform mole	Serial β-hCG monitoring (frequency not reported)
Tanudisastro et al., 2023 [16]	Case Report	30-year-old **β-hCG**: 15,156 IU/mL **Presenting Symptom**: right lower abdominal cramping, vaginal discharge	2 Right adnexal masses, one 3 cm and one 11 cm. 11 cm mass was anechoic with thick walled vascularity	Right salpingectomy	Complete hydatidiform mole	Serial β-hCG monitoring “per local protocol” (frequency not reported)
Toal et al., 2021 [17]	Case Report	31-year-old **β-hCG**: 103,724 mIU/mL **Presenting symptom:** vaginal bleeding **PMH**: G4P2012, uncertain LMP	31 × 43 × 31 mm mass arising from the right cornua containing echogenic internal debris and significant peripheral vascular flow	Laparoscopic right cornual wedge resection and right salpingectomy	Gestational trophoblastic neoplasia- stage 1 Invasive mole	Methotrexate and follow-up β-hCG measurements (frequency not reported) β-hCG = 0 at 3 month follow-up

The results reported here are similar to those reported in the literature review published by D'Asta (2022), particularly the age of onset, abdominal pain and vaginal discharge as the most common presenting symptoms, incidence of complete and partial moles and serial β-hCG monitoring as the primary mode of follow-up [7]. This review does report a higher incidence (28 %) of invasive gestational trophoblastic disease including choriocarcinoma than that of D'Asta (1/14 cases, 7 %). Of note, D'Asta reported 14 cases in one decade (1 January 2010 to 1 February 2021), and this report identifies an additional 13 in the following five years (1 September 2019 to 1 March 2024), including this case study, suggesting either an increase in disease frequency or increased reporting.

## 4 Discussion

Molar pregnancies are rare gestations resulting from abnormal fertilization and occur much less frequently than ectopic pregnancies [1]. Although exceptionally rare, molar ectopic pregnancies are clinically indistinguishable from nonmolar ectopic pregnancies and similarly treated with surgical or medical management based on appropriate β-hCG levels. Follow-up management, however, differs depending on the surgical specimen pathology.

Histopathological examination remains the gold standard for diagnosing GTD, but several reports of over-diagnosis based solely on histological examination have been described [17]. One study found only eight of 132 referred samples had a definitive diagnosis of HM and another study determined only three of 25 referred tubal ectopics had the histological findings of true molar pregnancy [17,18]. Given these discrepancies, ancillary studies, such as karyotype analysis, SNP array, p57 immunohistochemical staining, DNA flow cytometry, and molecular genotyping with polymerase chain reaction [4], should be utilized to confirm the diagnosis. This is critical because the risk of malignant GTN varies between 0.5 %–6 % for PHM and 15–20 % for CHM [6,17]. In fact, 4 (29 %) cases in this review presented with ectopic pregnancies arising from GTN that required methotrexate or other chemotherapy [9,12,16]. These findings underscore the importance of histological evaluation for all ectopic pregnancies to assess malignancy risk and determine appropriate follow-up [17]. In cases of GTD, β-hCG levels should be monitored weekly after surgery until undetectable, followed by monthly serum levels for 3 months in complete HM and 1 month for partial HM, which was done for the patient presented in this case study [6,19].

Additionally, discussions surrounding contraception are critical with all patients, and some form of effective contraception should be used until β-hCG levels normalize to distinguish between GTD and a new pregnancy [20]. Currently, all methods of contraception are acceptable after GTN, though caution should be used with intrauterine devices (IUDs) due to the theoretical risks for perforation, infection, hemorrhage, early discontinuation, and device expulsion [20]. Limited evidence suggests that women using an IUD after uterine evacuation for a molar pregnancy are not at increased risk for post-molar trophoblastic disease than are women using other methods of contraception [21]. Once β-hCG levels have decreased to nonpregnant levels, the risk for disease progression is likely to be very low [21].

Preoperative diagnosis of molar ectopic pregnancies remains challenging. There is insufficient evidence that ultrasonography (US) is a useful tool for distinguishing an ectopic tubal pregnancy from tubal molar pregnancy as there are no established criteria for this task, despite one report of ultrasonography used to support a diagnosis [7]. However, recent case reports suggest magnetic resonance imaging (MRI) may improve diagnostic accuracy in stable patients [14]. By using the high resolution of MRI, molar tissue can be identified as “heterogeneously enhancing cystic lesions” and may also show evidence of local invasion and vascularity that suggests a molar gestation [14]. In another report, a preoperative MRI diagnosis was made of molar ectopic pregnancy of the right uterine cornu [22]. Nonetheless, MRI is not cost-effective and is of limited practical utility in the evaluation of an ectopic pregnancy. While it should not be used routinely, MRI may be considered as a diagnostic adjunct in select complex cases in which tubal molar pregnancy is suspected (i.e. β-hCG out of proportion to gestational age or clinical expectation). This may allow for more informed preoperative counseling and planning.

## 5 Conclusions

Histopathologic evaluation and confirmatory lab techniques (i.e. immunohistochemistry, flow cytometry, molecular analyses) are essential for diagnosing molar ectopic pregnancies, as they determine a patient's risk for malignancy and guide post-operative management. Occasionally, malignant conversion can occur by the time of presentation as evidenced by the 29 % (4/14) of ectopic pregnancy secondary to GTN identified in this literature review. For the presented case, an isthmic tubal partial molar pregnancy was treated surgically and serial β-hCG levels were taken with no additional administration of chemotherapeutic agents. For select cases, MRI could provide a preoperative diagnosis of molar ectopic pregnancy.

## Contributors

Richard Q. Vuong contributed to patient care, conception of the case report, acquiring and interpreting the data, drafting the manuscript, and revising the article critically for important intellectual content.

Molly Hurd contributed to acquiring and interpreting the data, undertaking the literature review, drafting the manuscript, and revising the article critically for important intellectual content.

Zeynep Tek contributed to patient care, acquiring and interpreting the data, drafting the manuscript, and revising the article critically for important intellectual content.

Nicole Brzozowski contributed to patient care, conception of the case report, drafting the manuscript, and revising the article critically for important intellectual content.

All authors approved the final submitted manuscript.

## Patient consent

Written informed consent was obtained from the patient for publication of the case report and accompanying images.

## Provenance and peer review

This article was not commissioned and was peer reviewed.

## Funding

No funding from an external source supported the publication of this case report.

## Declaration of competing interest

The authors declare that they have no competing interest regarding the publication of this case report.
